# Prevalence and associated factors of thrombocytopenia among HAART naive HIV positive patients at Gondar university hospital, northwest Ethiopia

**DOI:** 10.1186/1756-0500-7-5

**Published:** 2014-01-06

**Authors:** Yitayih Wondimeneh, Dagnachew Muluye, Getachew Ferede

**Affiliations:** 1 School of Biomedical and Laboratory Sciences, College of Medicine and Health Sciences, University of Gondar, P.O. Box 196, Gondar, Ethiopia

**Keywords:** Thrombocytopenia, HIV, HAART naive, CD4 count

## Abstract

**Background:**

Hematological abnormalities are common in HIV positive patients. Of these, thrombocytopenia is a known complication which has been associated with progression of disease. However, its magnitude and associated factors in HAART naive HIV positive patients is not known in Ethiopia. Therefore, the aim of this study was to determine the prevalence and associated factors of thrombocytopenia in HAART naïve HIV positive patients.

**Methods:**

A retrospective study was carried out among HAART naive HIV positive patients at Gondar University Hospital, Northwest Ethiopia, from September 2011 through August 2012. Socio-demographic variables and immunohematological (platelets and CD4+ T cells) values were carefully reviewed from medical records. Associated factors and outcomes were assessed using logistic regression.

**Results:**

A total of 390 HAART naive HIV positive patients with a mean age of 33.65 years and a range of 18–70 years were reviewed. The overall prevalence of thrombocytopenia was 23(5.9%). The mean CD4 count was 288 ± 188.2 cells/μL. HIV patients whose age ≥ 50 years old were 2.5 times more likely to have thrombocytopenia and those patients whose CD4 count < 350 were 2.6 times more likely to have thrombocytopenia than HIV patients whose CD4 count ≥500. However, CD_4_ count was not statistically associated with prevalence of thrombocytopenia (P > 0.05).

**Conclusion:**

As CD4 counts of HIV patients decreasing, they have more likely to have thrombocytopenia. Therefore, early diagnosis and treatment of thrombocytopenia in these patients are necessary.

## Background

Human immunodeficiency virus (HIV) is a retrovirus that infects cells of the immune system, destroying or impairing their function, which leads to the occurrence of opportunistic infections and tumors [1]. Though the malfunction of the immune system and the decrease in the number and activity of CD4+ T cells signify the hallmark of HIV infection, it is notable that HIV can also impede with other cell lineages and tissues [2,3]. In addition to progressive reduction of CD4+ T cells, peripheral blood cytopenias, such as anaemia, neutropenia and thrombocytopenia, happen in most patients with AIDS [4,5].

Peripheral blood cytopenias have been showed even in the absence of chemotherapeutic treatment or opportunistic infections and tumours, signifying that HIV infection may be directly associated with the induction of these hematological abnormalities [6]. Intriguingly, identified thrombocytopenia can signify the first clinical manifestation in otherwise asymptomatic HIV positive patients [7] while neutropenia and anaemia are more common in the late stages of HIV disease [8].

Thrombocytopenia is characterized by platelet counts below 125 × 10^3^/mm^3^, and also frequently occurs in HIV-infected patients [9-11]. Its pathogenesis has not yet been recognized. Possible mechanisms that have been reported are increased platelet destruction, either caused by the non-specific deposition of circulating immune complexes on platelets or by the presence of specific anti-platelet antibodies, as well as direct infection of megakaryocytes by HIV with a resulting ineffective in platelet production [12].

Incidence of thrombocytopenia is around 40% of HIV-infected persons, and in approximately 10% of the patients, it may be the first sign of AIDS [13]. This haematological disorder may represent the first manifestation of HIV infection and it may progress over time and lead to severe bleeding [14]. Mature Megakaryoctes (MKs) can be infected by HIV through binding the CD4 receptor [15], and HIV genomes have been detected in MKs purified from bone marrow (BM) of HIV-positive patients [16].

The infection of MKs is not strain-restricted because both R5- and X4-tropic HIV-1 strains are able to infect MKs thus indicating that the infection may occur early in the development of HIV infection [17]. In addition to these direct effects of HIV on the MK cell lineage, HIV also supports chronic thrombocytopenia through autoimmune mechanisms [14], particularly manifest in early stages of the disease [18]. Autoimmune mechanisms are associated to anti-HIV antibodies cross-reacting with platelet-membrane glycoproteins, supporting the basic role of molecular mimicry in the induction of these antibodies [19].

Thrombocytopenia is associated with increased morbidity and mortality, accelerated deterioration in CD4 counts and accelerated progression to AIDS [20]. The incidence of thrombocytopenia varied according to the definition of thrombocytopenia and the characteristics of the baseline population [21]. There is no such information for HIV-infected individuals in Ethiopia which may help to inform respective bodies for treatment of HIV-infected individuals in this area. We therefore assessed the prevalence of thrombocytopenia in HIV-infected HAART naive patients and also tried to determine the relationship between thrombocytopenia and CD4 cell counts in these patients.

## Methods

A retrospective study was carried out among HAART naive HIV positive patients at Gondar University Hospital, Northwest Ethiopia, from September 2011 through August 2012. Gondar University Hospital provides HIV/AIDS interventions including free diagnosis, treatment and monitoring. The center diagnoses new cases and monitors those on therapy. Patients on interferon therapy, chemotherapy, or malignancy were excluded.

Socio-demographic variables and immunohematological (platelets and CD4+ T cells) values were carefully reviewed retrospectively from medical records. Reviewed laboratory data of platelets analyses were done using the automated blood analyzer Cell-Dyn 1800 (Abott Laboratories Diagnostics Division, USA) and CD4 T lymphocyte counts were done using the Becton Dickinson FACS count. Thrombocytopenia was defined as platelets count <125.0 × 10^3^/mm [22,23].

SPSS version 16 statistical software was used for analysis of the data. Descriptive statistics (minimum, maximum, mean and standard deviation) were used for continuous variables in the course of analysis and categorical data were analyzed using logistic regression. A P-value of < 0.05 was considered to be statistically significant.

Ethical clearance was obtained from the Institutional Ethical Review Board of University of Gondar and Permission for data collection was also obtained from the University Hospital.

## Result

A total of 390 HAART naïve HIV positive patient results were reviewed from their medical records. Out of these, 271 (69.5%) were females. The mean age of the patients was 33.65 ± 9.1 years, ranging from 18–70 years. The majority of study participants 328 (84.1%) were urban residents (Table 1).

**Table 1:** Socio-demographic characteristics of HAART naive HIV positive patients at Gondar university hospital, northwest Ethiopia, 2013

**Variables**	**Frequency**	**Percentage (%)**
**Sex**		
Male	119	30.5
Female	271	69.5
**Age in years**		
18-29	125	32.1
30-39	165	42.3
40-49	74	19
50 & above	26	6.7
**Residence**		
Urban	328	84.1
Rural	62	15.9
**Religion**		
Christian	358	91.8
Muslim	30	7.7
Others	2	0.5

The overall prevalence of thrombocytopenia was 23(5.9%). Platelet levels of the study participants were between 29×10^3^ cells/μl and 653×10^3^ cells/μl with the mean of 258×10^3^ ± 100.561 cells/μl. The minimum CD4 count was 9 cells/μL, and the maximum was 1280 cells/μL. The mean CD4 count was 288 ± 188.2 cells/μL. In this study, majority of the thrombocytopenia cases 12 (7.3%) were observed in the age group of 30–39 years. However, the difference was not statistically significant. HIV patients whose age ≥ 50 years old were 2.5 times more likely to have thrombocytopenia and those patients whose CD4 counts < 350 were 2.6 times more likely to have thrombocytopenia than HIV patients whose CD4 count ≥500 (Tables 2 and 3).

**Table 2:** Prevalence of thrombocytopenia among HAART naive HIV positive patients at Gondar university hospital, northwest Ethiopia, 2013

**Platelet count**	**Frequency**	**Percentage (%)**
≥125.0 × 10^3^/mm	367	94.1
<125.0 × 10^3^/mm	23	5.9
Total	390	100

**Table 3:** Association of thrombocytopenia with related factors among HAART naïve HIV positive patients at Gondar university hospital, northwest Ethiopia, 2013

**Variables**	**Thrombocytopenia**		**OR (95% CI)**		**P- value**
	**Yes (%)**	**No (%)**	**Crude**	**Adjusted**	
**Sex**					0.49
Male	9(7.6)	110(92.4)	1	1	
Female	14(5.2)	257(94.8)	0.7(0.28-1.58)	0.7(0.30-1.79)	
**Age**					0.46
18-29	5(4)	120(96)	1	1	
30-39	12(7.3)	153(92.7)	1.9(0.65-5-49)	1.7(0.57-5.06)	
40-49	3(4.1)	71(95.9)	1.0(0.24-4.37)	0.8(0.19-3.81)	
≥50	3(11.5)	23(88.5)	3.1(0.70-14.02)	2.5(0.54-11.66)	
**CD4 count**					0.57
<350	19(6.8)	260(93.2)	3.14(0.41-24.08)	2.6(0.33-20.61)	
350-499	3(4.5)	64(95.5)	2.02(0.20-20.02)	1.8(0.17-17.89)	
≥500	1(2.3)	43(97.7)	1	1	

Note: 1- reference group.

## Discussion

Increasing the intricacy of HIV infection, varied hematological manifestations can be seen, in which HIV related thrombocytopenia is one of them [24]. We assessed platelet counts in HAART naive HIV infected patients, which showed that 5.9% of the sample population had thrombocytopenia, which was in agreement with previous studies by Denue *et al*., [25], Sloand *et al*., [21], Sullivan *et al.,*[26], and Suresh *et al*., [27]. However, lower than reported by Erhabor *et al.,*[28] and Akinsegun *et al*., [29]. This problem is truly a medical challenge in vulnerable population, especially by the limited therapeutic options and the absence of intervention protocol for HIV subjects [9].

Results from this study showed that majority of HAART-naïve HIV positive patients were females. The female prevalence in this study confirms the World Health Organization (WHO) report that HIV/AIDS affects females most severely in sub-Saharan Africa [30,31]. However, thrombocytopenia had not showed statistical significance with sex and age (p > 0.05). This was in agreement with previous study done by Majluf-Cruz [24].

According to the present study, as immunity of a patient decreasing, thrombocytopenia was more prevalent rather than HIV positive patients who have relatively high CD4 count. For example, the prevalence of thrombocytopenia was proportionally high among patients who had a CD4 lymphocyte count of ≤ 350 cells/μL and low among patients with a CD4 count > 500 cells/μL. However, the increase in prevalence of thrombocytopenia with decreased CD4 cell count was not statistically significant (P >0.05). Similar findings have been reported by Elisaphane *et al.,*[32], Ira and Bhushan [33].

To the best of our knowledge, this is the first study in Ethiopia to determine prevalence of thrombocytopenia in HAART-naïve HIV infected patients. However, this study had limitations such as its retrospective nature which introduces possible biases related to ascertainment, documentation and chart review. In addition, this study was not included HIV infected patients who had on HAART. However, the observed results may still be a good reflection of a true circumstances and this study serve as a reference for additional recommendations to improve care of HIV infected persons and a step for further studies on the pathophysiology of HIV associated thrombocytopenia.

## Conclusion

In conclusion, as CD4 counts of HIV patients decreasing, they have more likely to have thrombocytopenia. Based on this finding, it is recommended that physicians giving care for HIV infected individuals should routinely investigate and treat thrombocytopenia.

## Competing interests

The authors declared no conflicts of interest with respect to the authorship and/or publication of this article.

## Authors’ contributions

YW: Participated in the conception and design of the study, data collection, analysis and interpretations of the findings, reviewed the manuscript. DM: Participated in the conception and design of the study, analysis and interpretations of the findings, reviewed the manuscript. GF: Participated in the conception and design of the study, data collection, analysis and interpretations of the findings, drafting the manuscript and write up. All authors read and approved the final manuscript.
